# Progress in Medicine

**Published:** 1896-07-11

**Authors:** 


### Progress in Medicine.

### DISEASES OF THE BLOOD.

**Pernicious Anæmia and Chlorosis.**—W. Hunter[1] insists that these are two totally different diseases, and that the former is not, as Stockman supposes, an extreme form of anæmia in which frequent internal hæmorrhages have occurred. He denies the frequency of the extravasations, or that any traces of them are found in the glands, and argues that in absorption of extravasated blood the increase of pigment deposited is found chiefly in the spleen, while in pernicious anæmia the liver is the chief seat. Still, the richness of these deposits show that the disease is due to blood destruction, unlike the anæmia of cancer or chlorosis, which occurs within the portal circulation, being caused by some ptomaine absorbed from the intestine. This poison is probably due to some special and rare organism. Ewald,[2] in confirmation of this view, mentioned a case when the iron in the liver amounted to ·693 per cent., instead of the normal ·078 per cent., and remarks that pernicious anæmia is often caused in Russia by tapeworm, probably through some toxin given off by the parasite. Stockman,[3] in reply, points out that in health the liver always keeps a reserve of iron to make up any sudden loss. The daily intake from food and the daily excretion are each only about $\frac{1}{4}$ grain, and the total iron in the body 38 grains, of which the liver contains $3\frac{1}{2}$, while the iron in the spleen is much less, though variable. With regard to diseases where severe anæmia occurs without external hæmorrhage, he shows by analysis of a series of cases that the iron in the liver is much increased, while that in the spleen varies as in health. If external hæmorrhage is the cause, the iron in the liver is diminished. There is no difference between pernicious and other anæmias in this respect, nor in the excess of yellow colouring matter in the skin, nor does he think the absorption of a ptomaine at all proved. Two cases of pernicious anæmia have been recently reported by Hayem.[4] In one of the two gastric atrophy was present, and in the other gastritis, but there are great numbers in which no such lesions are present, so that it is doubtful whether gastric disease is more than an occasional cause.

A detailed account of three cases treated with bone marrow under Dr. Ringer is given by G. B. Hunt. There was a relative excess of hæmoglobin, and much poikilocytosis, no spinal symptoms, but in two of them hæmorrhages under the tongue. The bone marrow had no effect. The author, collecting the results of marrow treatment, finds two cases only recorded as showing benefit, while in eight others it failed. He concludes that marrow should only be administered in cases where treatment by arsenic has failed. Menella[5] has had rapid improvement under injections of iron and iodine. He injects at the same time citrate of iron and ammonium, and also reduced iron with iodide of potash in different situations. Stockman[6] has continued his analytical study of chlorosis, and attributes it to an insufficient supply of iron in the food, and often to some preceding loss of blood. It has been shown by urinary and intestinal analysis that Sir A. Clark's theory of the absorption of ptomaines is to say the least unsupported by facts, and that Bunge's sulphuretted hydrogen explanation is equally baseless. Again, though there is no evidence of the breaking up of red corpuscles in chlorosis, and they are not always below the normal in number, yet there is always a deficiency in hæmoglobin. Now, this is just the condition which is caused by feeding an animal on food deficient in iron, and numbers of chlorotic patients are dyspeptics, and narrowly limit their diet on account of pain. Moreover, the analysis of the food of certain chlorotic patients showed that it actually contained only a third of the normal amount of iron. The loss of iron from menstruation in such patients cannot be made up, and the amenorrhœa of fully-developed chlorosis is probably a protective action, for as much iron is lost in one menstruation as a healthy person gets from food in a week. Thus, insufficient food, menstruation in adolescent persons, and possibly in some cases feeble blood-forming power, are the causes of chlorosis. The treatment should consist in giving iron in any one of the easily-taken forms, in improving the appetite and digestion, and checking undue loss. Proofs of the utilisation of inorganic iron by the organism, in opposition to Bunge's denial of it last year, have flowed in from many quarters. Thus, Woltering,[7] of Utrecht, shows that regeneration of red corpuscles is much more rapid in animals who have suffered severe hæmorrhage if their food is mingled with iron. Nor does the inorganic iron merely combine with the sulphuretted hydrogen of the intestine, and so shield the organic iron in the food, for manganese would combine equally well with the $H_2S$, but it causes no increase of iron in the liver, and this the administration of iron does do. In fact, the iron is there built up into organic compounds. Kunkel[8] asks whether these compounds are identical with the iron normally stored there, and whether the hæmoglobin is formed from this absorbed iron. To settle this he fed two dogs on food almost free from iron, and bled them every week. To one he administered iron daily. It remained free from anæmia, though ·36 grammes of iron had been removed in the bleedings. After death the analysis of the blood and the tissues showed a very large excess of iron over that of the other dog, the amount in the liver being especially great. The rapidity of the regeneration of the blood was remarkable, for in seven weeks more than twice the blood in the body had been removed by bleeding. Deutsch and others[9] testify to the great value of ferratin in anæmia, especially in that resulting from gastric ulcer, where the stomach is very irritable, and in cases where treatment with other iron salts has failed. G. T. Richardson[10] gives it in eight-grain doses three times daily with great success, and finds that it is well borne in irritable stomachs. It is particularly useful for anæmic infants.

Three fatal cases of thrombosis of the cerebral sinuses in chlorosis have been reported by Lee Dickinson,[11] who remarks that it occurs more often in chlorosis than in any other disease. Chlorotic blood not infrequently forms thrombi in the veins of the legs, and the rarity of thrombi in the sinuses depends probably on the small amount of $CO_2$ in the cerebral blood. Lesser degrees of sinus thrombosis are not always fatal, and are perhaps more common than we suppose. The chief symptoms are optic neuritis and severe headache. Two other cases were mentioned by Dyce Duckworth, one of them being in a woman suffering from white leg. He recommended iron with alkalies and the free use of alcohol and ammonia. Three other instances were referred to in the discussion. In one, convulsive attacks, supposed to be hysterical, came on, but the patient gradually recovered under the use of iron, and the optic neuritis disappeared. The affection of the spinal cord in pernicious anæmia is by no means the same as that in locomotor ataxy. T. H. Lloyd[12] describes the pathology of a case with great fulness, and refers to previous investigations. It is essentially a type of posterior sclerosis, in which the root zone and Lissauer's tract escape. There may be degeneration also of the crossed and direct pyramidal tracts, and the earlier process appears to be a swelling of the medullary sheaths. Variation in the areas affected may occur with more or less ataxic symptoms. He is inclined to regard the whole process as one caused by some toxin which first attacks the myeline sheaths, and in connection with this he remarks upon the high temperature and diarrhœa which occurred in his patient.

**Leucocythemia.**—The acute form is discussed by Fränkel,[13] who has met with nine cases. It commences suddenly in healthy persons with anæmia, syncope, hæmorrhages, swelling of glands, and enlargement of the liver and often of the spleen. Pyrexia appeared in half the cases, and the bone marrow was always affected; the duration was from three to sixteen weeks. There was a diminution of the red corpuscles and an increase of the mononuclear leucocytes. The number of eosinophile and polynuclear corpuscles was not increased. In the lymphatic glands the same increase was observed with respect to the mononuclear bodies. The author believes that the blood changes are essentially the same in this disease as in chronic lymphocythemia, only that the newly-formed elements here pass with great rapidity into the blood-stream before they have had time to undergo any change. In chronic leucocythemia Buchanan[14] finds two types of blood changes—(1) where forms of cells not usually seen in normal blood appear. There are many large eosinophile cells with coarse granules, and large uninuclear cells. In (2) there are many small lymphocytes and some large hyaline ones, as well as transitional forms in both. G. L. Gulland, in the "Proceedings of the Royal Society,"[15] describes all leucocytes as merely stages in the development of a tissue. The earliest stage is a lymphocyte from which small hyaline forms arise. They may remain hyaline or develop into acidophile or basophile types. The granules are not products of the metabolism of the cell, but represent an altered condition of the mitoma, and are probably concerned with amœboid movement. The more active the cell the more visible are they, and the rest of the mitoma. T. Henrick[16] points out that we may be misled in our diagnosis of leucocythemia, especially if the patient has been under treatment, and we happen to find the proportion of white to red corpuscles normal, unless we stain the blood and discover the myelocytes; or, again, an increase in white corpuscles may be from leucocytosis as in pneumonia, but in this case the increase in the cells will be from polynuclear neutrophiles, and there will be no enlargement in the spleen and glands. If the increase be in small nucleated cells, and the glands are enlarged, we have to do with lymphatic leukœmia; and finally, if besides the increase in large mononuclear leucocytes, characteristic of ordinary leucocythemia, we find an increased number of eosinophilous cells, then we have to do with splenic leucocythemia. Thus, even without staining, an increase of large cells means splenic or ordinary leucocythemia; an increase of small ones lymphocythemia, if in either case we can exclude leucocytosis.

**Septicæmia.**—Grawitz[17] finds usually a high degree of leucocytosis in septicæmia, *i.e.*, of polynuclear neutrophiles, and mentions that in this disease there is a great progressive reduction of the dry residue of the blood, amounting to about 1 per cent. daily out of a normal of 21 per cent. The enormous loss of albumen is also shown by the reduction of the dry residue of the serum. The hæmoglobin of individual red corpuscles is not lessened, but their number often is, yet the great thinning of the blood is chiefly due to transudation from the tissues, caused by the toxins. Finally, in every case where the dry residue of the blood was reduced by one-third, a fatal result followed.

Denham and M'Weeney[18] recently showed a case of splenic leucæmia which was peculiar in that no large mononuclear cells could be found. This was, they thought, a favourable point in the prognosis. Köster[19] discusses a case of true splenic anæmia, where no increase of white cells existed, but the red were enormously reduced, and many nucleated and otherwise degenerate corpuscles occurred. The spleen was slightly enlarged. Under arsenic and quinine the patient got worse, until inhalations of oxygen were tried with success. The arsenic was also continued for some time. In splenic leucocythemia a case is recorded by J. R. Whait which[20] recovered for a time, and showed normal blood contents, under bone-marrow treatment, but died from a lung complication. There had been some bronzing of the skin, and atrophy of the suprarenal bodies was found post-mortem.

**Lymphadenoma.**—Stephen Mackenzie[21] records an instance in which the first symptoms were those of "double sciatica"; then followed epistaxis, anæmia, pyrexia, enlarged glands in the neck and groin, with a red, tender, œdematous area over the sacrum. This was found post-mortem to be due to a lymphadenoma which extended into the pelvis, and penetrated the spinal canal and the two sacro-iliac joints. Other tumours were present, and the disease rapidly progressed to a fatal termination. In a second case the symptoms began with sciatic pains; then followed emaciation, enlarged glands, and much pain in the stomach, which led to the suspicion of cancer of the stomach. A lymphadenoma was found adherent to the vertebræ, binding down the stomach, and invading the spleen. In appearance it was very like scirrhus. J. S. Ely[22] records a third case, commencing with pain in the dorsal and sciatic region. The growths were confined to the posterior mediastinal glands, the lungs, and the liver. Unlike Mackenzie, he holds that the growths do not encroach upon surrounding tissues, and cannot be regarded as akin to sarcoma. A patient of F. S. Mandelbaum[23] suffered from a similar growth, which occluded the thoracic duct, and caused chylous ascites and chylous hydrothorax. Subcutaneous injections of arsenic are said by Moritz and Ziemssen to have cured cases of lymphadenoma, and the latter author[24] finding that Fowler's solution gives pain when injected, prepares an injection of sodium arseniate, of which doses rising to three-tenths of a grain of the drug are given daily without inconvenience. G. Francesco[25] claims satisfactory results in some forms of anæmia from the injection of bichloride of mercury and bichloride of quinine. Whether his cases were chlorosis or pernicious anæmia is not clear from his description. C. G. Stockton[26] refers to the diminished alkalinity of the blood in severe anæmia, and the great amount of uric acid in the water, as well as the practical importance of treating this by sufficient alkalies before administering iron. He believes that in some cases they may be substituted for iron. The whole question of the hypodermic administration of iron and its advantages is well discussed by Da Costa.[27] He personally prefers the ferrous manganese citrate, or the iron and ammonium citrate, in doses of one to five grains daily, but mentions a dozen other preparations which have been employed. The method is valuable where rapid action is desired, as, after severe hæmorrhages, in gastric ulcer, obstinate constipation, or where we desire to act specially on the nervous system. The excess of iron seems to remain as a store in the system, possibly in the liver, as it is not easily excreted, and has probably much more lasting effects than when given by the mouth. Stengel[28] insists on the value of massage as well as of drugs in the treatment of severe anæmias to equalise the distribution of the red corpuscles, and to return to the circulation those which are stored away in the glands. Since the available corpuscles are so few, it is of importance to drag into use all we have. The same action is said to be brought about by the use of cold baths in typhoid fever. Again, if the quantity of blood in the body is scanty we must employ rectal or venous injections of saline fluid to increase it if we wish to see any benefit from the iron or arsenic we give. This he thinks of special importance in pernicious anæmias, where, too, he employs intestinal lavage and antisepsis.

[1] B.M.J., Feb. 8, 1868. [2] Med, Week., March 6. [3] B.M.J., May 2. [4] Med. Week., April 3. [5] Med. Week., Nov. 22, 1895. [6] B.M.J., Dec. 14. [7] Med. Week., Mar. 27. [8] Ther. Gaz., March 16. [9] B.M.J., Jan. 11. [10] N.Y. Med. J., April 18. [11] B.M.J., Jan. 18. [12] J. of Nervous and Ment. D., April. [13] Med. Chron., Jan. [14] Lancet, Jan. 25. [15] N.Y. Med. J., Feb. 8. [16] Internl. Clinics, iv., 2. [17] Ditto. [18] B.M.J., Feb. 1. [19] Practit., April. [20] B.M.J., April 4. [21] Lancet, Jan. 4. [22] Med. Rec., Feb. 24. [23] Med. Rec., Feb. 8. [24] Practit., Jan. [25] B.M.J., April 11. [26] Internl. Clinics, iv., 2. [27] Ther. Gaz., May. [28] Ibid.

